# Inflammatory Response in COVID-19 Depending on the Severity of the Disease and the Vaccination Status

**DOI:** 10.3390/ijms24108550

**Published:** 2023-05-10

**Authors:** Felicia Trofin, Eduard Vasile Nastase, Manuel Florin Roșu, Aida Corina Bădescu, Elena Roxana Buzilă, Egidia Gabriela Miftode, Doina Carmen Manciuc, Olivia Simona Dorneanu

**Affiliations:** 1 Microbiology Department, University of Medicine and Pharmacy “Grigore T. Popa”, 700115 Iasi, Romania; felicia.trofin@umfiasi.ro (F.T.); aida.badescu@umfiasi.ro (A.C.B.); olivia.dorneanu@umfiasi.ro (O.S.D.); 2 Clinical Hospital of Infectious Diseases “Sfânta Parascheva”, 700116 Iasi, Romania; eduard-vasile.nastase@umfiasi.ro (E.V.N.); manuelflorin.rosu@gmail.com (M.F.R.); dmanciuc@yahoo.com (D.C.M.); 3 Infectious Diseases Department, University of Medicine and Pharmacy “Grigore T. Popa”, 700115 Iasi, Romania; 4 Department of Dento-Alveolar Surgery, Anesthesia, Sedation, and Medical-Surgical Emergencies, University of Medicine and Pharmacy “Grigore T. Popa”, 700115 Iasi, Romania; 5 Iasi Regional Center for Public Health, National Institute of Public Health, 700465 Iasi, Romania; elenaroxanabuzila@yahoo.ro

**Keywords:** COVID-19, SARS-CoV-2, severity, IL-6, ferritin, LDH, CRP

## Abstract

The aim of this study was to analyze the serum concentration of interleukin-6 (IL-6), C-reactive protein (CRP), D-dimer, lactate dehydrogenase (LDH), ferritin, and procalcitonin in COVID-19 patients with different forms of the disease. We performed a prospective cohort study on 137 COVID-19 consecutive patients, divided into four groups according to the severity of the disease as follows: 30 patients in the mild form group, 49 in the moderate form group, 28 in the severe form group, and 30 in the critical form group. The tested parameters were correlated with COVID-19 severity. Significant differences were registered between the form of COVID-19 depending on the vaccination status, between LDH concentrations depending on the virus variant, and in IL-6, CRP, and ferritin concentrations and vaccination status depending on the gender. ROC analysis revealed that D-dimer best predicted COVID-19 severe forms and LDH predicted the virus variant. Our findings confirmed the interdependence relationships observed between inflammation markers in relation to the clinical severity of COVID-19, with all the tested biomarkers increasing in severe and critical COVID-19. IL-6, CRP, ferritin, LDH, and D-dimer were increased in all COVID-19 forms. These inflammatory markers were lower in Omicron-infected patients. The unvaccinated patients developed more severe forms compared to the vaccinated ones, and a higher proportion of them needed hospitalization. D-dimer could predict a severe form of COVID-19, while LDH could predict the virus variant.

## 1. Introduction

Cytokines are key factors in mediating COVID-19, in the pathogenesis and progression of the infection [1]. Cytokine secretion is triggered after the internalized virus contacts epithelial cells, macrophages, or dendritic cells [2]. There are cytokines that correlate with the severity of COVID-19, cytokines associated with disease onset, and cytokines that can be used as predictors of critical infection. Interleukin-6 (IL-6) is an indicator of the activation of the innate immune response. It increases rapidly until it reaches a peak at the onset of symptoms and begins to slightly decrease thereafter [3]. The overproduction of proinflammatory cytokines leads to high procalcitonin secretion [4]. The exaggerated inflammatory reaction, objectified by cytokine storm, macrophage, and endothelial activation, along with diffuse intravascular coagulation, immobilization of the patients, and hypoxia can result in venous thromboembolism. Infected patients are at risk for arterial and venous thrombosis, venous thromboembolism, deep vein thrombosis, and pulmonary embolism. The D-dimer level increases in patients with venous thromboembolism [5]. Although ferritin is known to be an acute-phase marker of inflammatory diseases, it is still unclear whether its role is to mediate inflammation or is a result of it. It can also provide information on the extent of acute or even chronic inflammation in many diseases [6]. Lactate dehydrogenase (LDH) isoenzymes are released after cell damage or apoptosis. They may signal vascular permeability in immune-mediated lung injury [7].

According to World Health Organization (WHO), there are persons who become infected with SARS-CoV-2 and remain asymptomatic and patients that become symptomatic. Most people develop mild or moderate COVID-19, some people develop severe disease that requires oxygen support, and fewer patients have critical disease with complications such as respiratory failure, acute respiratory distress syndrome, multi-organ failure, and thromboembolism [8].

Throughout the pandemic, several SARS-CoV-2 variants of concern appeared, due to the genetic variation of the virus over time. These emerging variants (Alpha, Beta, and Delta) harbor some mutations that may influence their behavior, with an impact on the virus transmissibility and pathogenicity [9]. In November 2021, a new variant of concern named Omicron was reported, and it triggered a fourth wave of COVID-19 that spread worldwide. The Omicron variant was associated with reduced severity and affected the younger population with a high rate of vaccination [10].

During the pandemic, hospitals were overcrowded, even reaching times when some patients could no longer have access to hospitalization. For a correct screening of patients with COVID-19 who may progress to respiratory failure and require hospitalization, the following criteria may be helpful: symptoms, oxygen saturation, respiratory rate, chest imaging, laboratory tests, and underlying medical conditions. It is important to note that these criteria are not exhaustive, and clinical judgment should always be used when evaluating patients with COVID-19. Categorizing patients based on their risk of developing severe or critical forms of COVID-19, even during their initial visit to the physician, can be lifesaving and also has an economic impact due to the triage of severe cases. Inflammatory markers, including IL-6, C-reactive protein (CRP), D-dimer, LDH, procalcitonin, and ferritin, together with the patient’s age and gender can be used as risk indicators. The change in the level of inflammatory markers can distinguish severe from mild/moderate COVID-19 cases [11]. Ultimately, the decision to hospitalize a patient should be based on a careful assessment of the patient’s clinical status and laboratory tests. Our aim was to detect and analyze the concentrations of the above-mentioned inflammatory markers according to the mild, moderate, severe, and critical forms of SARS-CoV-2 infection, the vaccination status, and the virus variant.

## 2. Results

**Figure 1:**
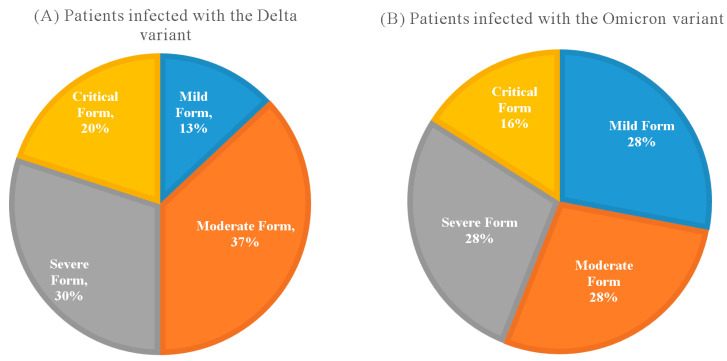
(**A**) Patients infected with the Delta variant and the severity of COVID-19 they have developed. (**B**) Patients infected with the Omicron variant and the severity of COVID-19 they have developed.

The study lot included 137 SARS-CoV-2-infected patients. Thirty (21.9%) of them had a mild form of COVID-19, forty-nine (35.8%) had a moderate form, twenty-eight (20.4%) had a severe form, and thirty (21.9%) had a critical form of the disease. Seventy-seven (56.2%) of the patients were men. Forty-five (32.8%) of the patients were vaccinated (Table 1). The PCR mutation detection revealed the Delta variant in sixty (43.8%) patients and the Omicron variant in twenty-five (18.2%) (Table 1) (Figure 1A,B); only eighty-five patients were tested due to economic reasons. The IL-6 mean concentration was 125.64 pg/mL (Table 1).

According to the Kolmogorov–Smirnov variable distribution test, all the variables are abnormally distributed. The Spearman test shows positive correlations between all serum parameters (except procalcitonin) and the severity of COVID-19 (*p* < 0.05, *r* > 0) (Table 2). All tested inflammatory marker concentrations correlated with each other, being directly correlated (*p* < 0.05) (Table 2).

Using the independent samples test, we compared the serum parameter concentrations depending on the disease form. In addition, we compared the biomarkers depending on the vaccination against SARS-CoV-2 status, virus variant, or gender. We performed the same evaluation for the form of COVID-19 by vaccination status, virus variant, and gender. Significant differences were registered between the form of COVID-19 depending on the vaccination status (*p* = 0.007), between LDH concentrations depending on the virus variant (*p* = 0.026), and in IL-6 (*p* = 0.039), CRP (*p* < 0.001), ferritin concentrations (*p* = 0.007), and vaccination status (*p* = 0.012) depending on the gender. There are significant differences in ferritin concentration between the mild and moderate forms of infection (*p* = 0.018). Differences were also recorded between the moderate and severe forms of the disease in IL-6 (*p* = 0.005) and CRP concentrations (*p* < 0.001). The critical form showed differences in the IL-6 (*p* = 0.048) and LDH concentrations (*p* = 0.027) compared to the severe form (Table 3).

**Figure 2:**
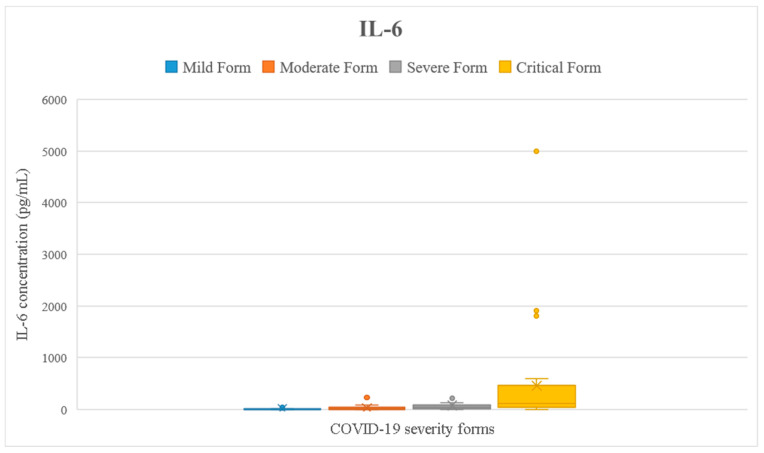
Interleukin-6 concentration depending on COVID-19 form for all the patients included in the study.

**Figure 3:**
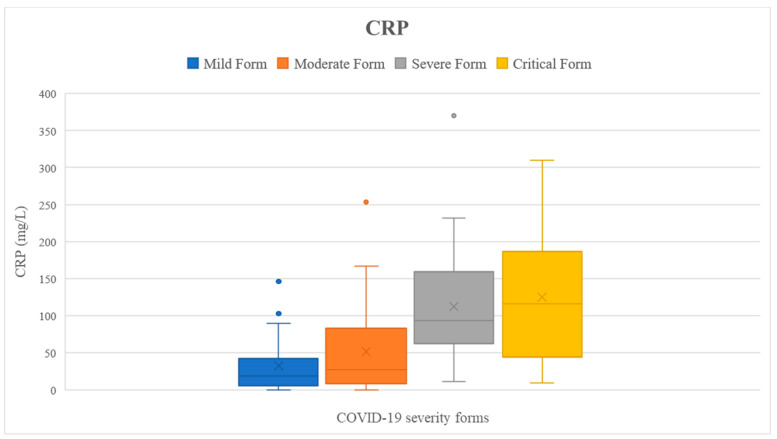
C-reactive protein concentration depending on COVID-19 form for all the patients included in the study.

**Figure 4:**
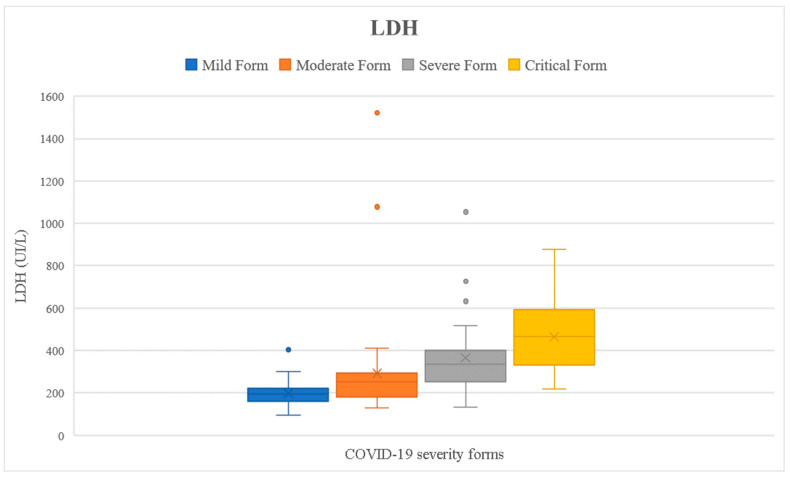
Lactate dehydrogenase concentration depending on COVID-19 form for all the patients included in the study.

**Figure 5:**
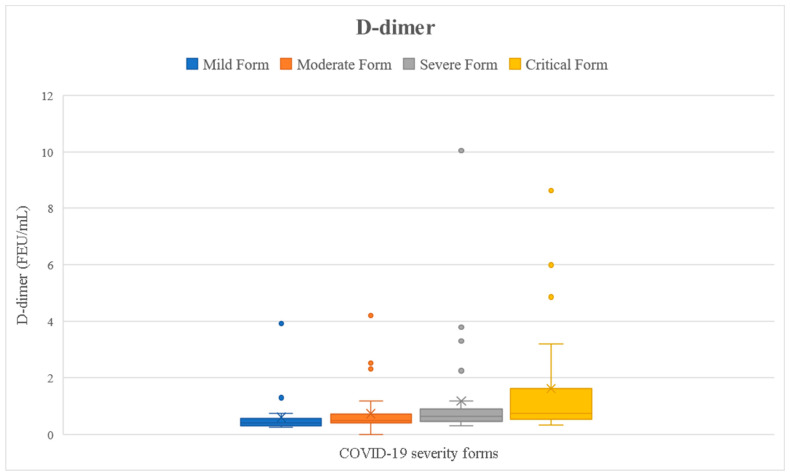
D-dimer concentration depending on COVID-19 form for all the patients included in the study.

**Figure 6:**
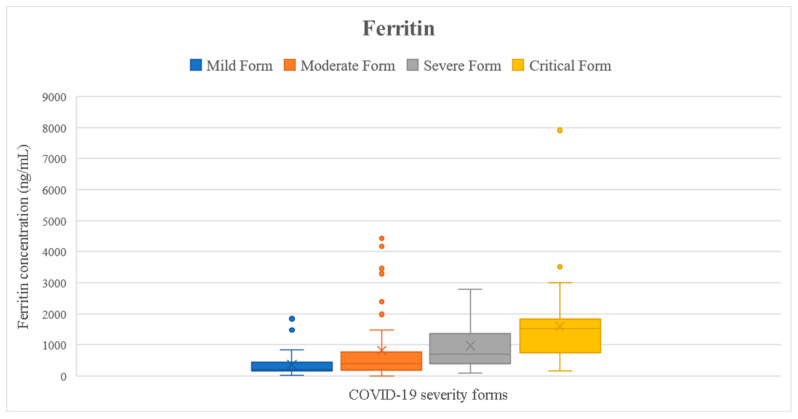
Ferritin concentration depending on COVID-19 form for all the patients included in the study.

We checked if the results of the tested parameters were significantly increased compared to the reference interval (presented in Table 1) using the one-sample *t*-test. Analyzing the entire cohort, we noticed that all inflammatory markers were significantly higher than the reference interval (*p* < 0.05) (Figure 2, Figure 3, Figure 4, Figure 5 and Figure 6). Analyzing the biomarkers within each study subgroup, we revealed that in mild COVID-19, only the CRP concentration was significantly increased, while in the other forms of COVID-19, all markers were significantly elevated (*p* < 0.05) (Figure 2, Figure 3, Figure 4, Figure 5 and Figure 6). In the mild form group of patients, only CRP was significantly increased, while in the other groups, IL-6, ferritin, LDH, and D-dimer were also significantly increased. The IL-6 concentration exceeded the reference range even for the mild forms of the disease (mean IL-6 = 11.96 pg/mL). It slightly increased in moderate forms (mean IL-6 = 25.46 pg/mL), for which the mean IL-6 concentration doubled compared to mild forms, and in severe forms (mean IL-6 = 78.03 pg/mL), for which the mean was 7 times higher compared to mild forms. In critical forms (mean IL-6 = 458.31 pg/mL), the IL-6 response far exceeds even the one in severe forms. This aspect was previously described by Trofin et al. (2023) [12]. The mean CRP value in mild COVID-19 patients (32.74 mg/L) was 4 times lower than the one in critical COVID-19 patients (129.06 mg/L).

Some important statistical parameters such as mean, median, standard deviation, and variance of the concentration of the tested biomarkers are available in Table 4.

The maximum concentration of IL-6 recorded in the critical form of COVID-19 of a vaccinated patient was 494.8 pg/mL, and the maximum concentration of IL-6 of an unvaccinated patient with the critical form was 5000 pg/mL. The highest value of CRP recorded in a vaccinated patient with the critical form was 148.06 mg/L, while the maximum CRP recorded in an unvaccinated patient with the critical form was 532 mg/L. The highest LDH value recorded in a vaccinated patient who developed a critical form was 597 UI/L vs. 878 UI/L, the value that was recorded in an unvaccinated patient with a critical form. The highest concentration of ferritin was registered in a vaccinated patient with critical COVID-19 (7915.9 ng/mL), and it was 2 times higher than the highest value recorded in a non-vaccinated critical patient (3509 ng/mL). The maximum D-dimer value obtained in vaccinated critical patients was 4.85 FEU/mL, while for the unvaccinated critical patients, this value was 8.63 FEU/mL (Figure 2, Figure 3, Figure 4, Figure 5 and Figure 6).

The infective variant was detected in 85 (62%) patients; 70.6% were infected with the Delta SARS-CoV-2 variant, and 29.4% were infected with the Omicron one. The highest proportion of the patients infected with the Delta variant had moderate COVID-19 (37%), closely followed by the severe form (30%) and the critical one (20%). Patients infected with the Omicron variant were equally distributed (28%) between mild, medium, and severe forms of COVID-19.

The procalcitonin value correlated only with the form of COVID-19. It did not register other statistically significant correlations with any other parameter.

We performed several ROC analyses in order to determine the sensitivity and specificity of the investigated inflammation parameters in predicting a severe form of COVID-19 or in predicting the infective SARS-CoV-2 variant. The biomarker that best predicted a severe form of COVID-19 was D-dimer, leading to an AUC value of 0.813. The corresponding cut-off value was 0.725 FEU/mL (sensitivity = 1.000; specificity = 0.350). The other tested parameters could not predict severe COVID-19 (Supplementary Materials Figure S1). The parameter that best predicted the virus variant was LDH, leading to an AUC value of 0.760. The corresponding cut-off value was 232 UI/L (sensitivity = 0.813; specificity = 0.333). A higher LDH value than the cut-off value most probably indicates an infection with the Delta viral variant. The other biomarkers could not predict the virus variant (Supplementary Materials Figure S2).

## 3. Discussion

In the current study, we analyzed the inflammatory response of 137 COVID-19 patients, hospitalized in the Clinical Hospital of Infectious Diseases “Sfânta Parascheva” Iași, during the fourth and the fifth waves of the COVID-19 pandemic. We chose to include patients with any severe form of COVID-19, aiming to compare the parameters according to the disease’s severity and the vaccination status prior to infection. All the vaccinated patients were vaccinated with two doses of a mRNA vaccine during 2021. We chose to analyze the CRP, LDH, ferritin, D-dimer, and procalcitonin in the serum collected on the first day of hospitalization and IL-6 on the 7th day of hospitalization. At the time of IL-6 collection, all patients were under treatment. None of the patients received tocilizumab until the time of collection of the serum sample intended for IL-6 analysis.

IL-6, CRP, LDH, ferritin, D-dimer, and procalcitonin concentrations increased as the severity of the disease increased (*p* < 0.05) (Table 2). The mean IL-6 concentration that we calculated for each of the four studied subgroups was in the range of values obtained by other authors (Table 5).

The tested inflammatory markers were significantly increased compared to the reference interval for all COVID-19 patients (Table 1). Our results are in line with Kleymenov et al. (2021), who found that IL-6 showed increased expression associated with disease severity [3].

IL-6, CRP, LDH, and ferritin are higher in men compared to women (*p* < 0.05). These results might be related to the fact that men more frequently show severe forms compared to women. Kleymenov et al. (2021) [3], Scully et al. (2020) [18], and Angioni et al. (2020) [19] suggested that IL-6 is a gender-associated cytokine in COVID-19 because males develop higher concentrations. The findings of Qin et al. (2020) [20] support our results, as they observed that male patients showed greater inflammation, with higher levels of LDH, ferritin, and CRP.

We demonstrated an increase in the procalcitonin concentration correlated with the severity of the disease; our results are similar to those of Mazaheri et al. (2022) [4], who concluded that procalcitonin was significantly higher in their intensive care unit cohort. In addition, as in our case, males in their cohort had higher procalcitonin levels compared to females.

We have performed a comprehensive analysis of scientific papers that aimed to analyze the values of D-dimer depending on the severity of the disease, and we noticed that our mean concentrations are similar to those of other authors (Table 6).

According to the systematic review and meta-analysis of Kaushal et al. (2022) [6], the serum ferritin level was significantly higher in the severe to critical patients compared to the mild to moderate category of patients in 39 of the analyzed studies. Our findings support these outcomes; the ferritin concentrations were similar to those from other studies, according to the severity of COVID-19 (Table 7).

In a meta-analysis, Fialek et al. (2022) [29] outlined the role of elevated LDH levels in assessing the severity of COVID-19. They observed lower LDH levels in mild and moderate groups compared to the severe course of COVID-19, and lower LDH levels in the severe group compared to the critical group. Our conclusions are similar to those described by Fialek et al. (2022) [29]. The mean values of LDH in the severe/critical forms of this infection are close to those published in other studies (Table 8).

CRP is a highly sensitive biomarker for inflammation, tissue damage, and infection [30]. This acute-phase protein is a relatively cheap way and within the reach of any laboratory for screening for these pathologies. It was shown in many studies that the CRP value increased in COVID-19 patients; in critical form patients, it reached values 5 to 10 times higher than those in non-severe patients [31,32,33,34,35,36,37]. The same findings were highlighted in our study. The inflammatory reaction in our patients was more important than that described by Sun et al. (2020) [13], Liu et al. (2020) [22], Shang et al. (2020) [23], and Zheng et al. (2020) [15], but it was in line with that described by Trofin et al. (2023) [12], Liu et al. (2020) [14], Wang et al. (2020) [27], and Chen et al. (2020) [28] (Table 9).

As only one-third of the patients included in the study were vaccinated against SARS-CoV-2 before the onset of the disease, we conclude that most hospitalized patients were unvaccinated. As in other studies, significant differences were recorded between the vaccination status and the form of COVID-19 developed by the patients (*p* < 0.05) [38,39,40,41,42]. Even if no significant differences were recorded, the non-vaccinated patients had a more important inflammatory response compared to the vaccinated ones.

Suzuki et al. (2022) [43] argued that LDH, CRP, ferritin, aspartate aminotransferase, and neutrophil-to-lymphocyte ratio levels were significantly lower in the Omicron group. In our study, patients infected with the Omicron strain had lower LDH values compared with Delta strain patients (*p* < 0.05).

According to ROC analysis, a D-dimer value higher than 0.725 FEU/mL could predict a severe form of COVID-19, and an LDH value higher than 232 UI/L could predict the result of the viral genome sequencing as the Delta variant.

### 3.1. Strength of the Study

The study considered all the forms of COVID-19, also describing the characteristics of patients with mild and moderate COVID-19. In addition, other variation factors were taken into account, such as the viral variant, the vaccination status, and the gender of the patients.

### 3.2. Limitations

Due to financial reasons, we could not establish the virus variant for all the patients. In addition, the virus variant was assessed following PCR mutation testing, not after viral sequencing. A larger sample size would increase the statistical power of the study and reduce the risk of type II error. The study includes participants who were admitted to a specific infectious disease hospital, which preferentially and mostly hospitalized COVID-19 patients from the Iași metropolitan area, which may not be representative of the broader population. This could limit the generalizability of the study’s findings. Confounding factors, such as age, comorbidities, and medications, could impact inflammatory marker levels and COVID-19 severity. The study has not accounted for confounding factors such as comorbidities, medications, or age, which could limit the validity of the findings. Measuring inflammatory markers at multiple time points throughout the disease course would provide a more comprehensive understanding of the relationship between inflammation and COVID-19 severity.

## 4. Materials and Methods

### 4.1. Study Design and Participants

We performed a prospective cohort study aimed to analyze the IL-6, CRP, D-dimer, LDH, ferritin, and procalcitonin concentrations of SARS-CoV-2-infected patients hospitalized in the Clinical Hospital of Infectious Diseases “Sfânta Parascheva” Iași between 26 October 2021 and 4 April 2022.

The inclusion criteria were as follows: a. age over 18 years old at the time of enrollment in the study; b. a positive SARS-CoV-2 RT-PCR test at admission to the hospital; c. hospitalization at no more than 10 days from the onset of symptoms; d. without tocilizumab administration until the time of serum collection, and the patient’s consent to participate in the study.

The exclusion criteria were as follows: a. patient’s refusal to participate in the study; b. lack of information regarding the onset of symptoms; c. insufficient amount of serum sample.

We included 137 consecutive patients who met the inclusion and exclusion criteria in the study. SARS-CoV-2 RNA detection was performed by RT-PCR on both oropharyngeal and nasopharyngeal swab samples. The patients were divided into 4 groups according to the severity of the disease as follows: 30 patients in the mild form group, 49 in the moderate form group, 28 in the severe form group, and 30 in the critical form group. We used the WHO criteria for assigning patients to a group of the severity of the disease [8]. The severity of the disease was assessed according to the clinical evolution of the patient during hospitalization. It was assessed upon discharge from the hospital, with patients having an average length of hospital stay of 10.4 days.

### 4.2. Collection and Analysis of Samples

We collected one serum sample for each of the enrolled COVID-19 patients at 7 ± 1 days after the declared onset of the symptoms. When choosing the sampling day, we took into account exclusively the onset of symptoms and not the date of admission in the hospital. Since all the patients were hospitalized, the samples were collected in the hospital, and they were immediately transported to the laboratory. The sera were stored in Eppendorf tubes at −80 °C until analyzed. We quantified the IL-6 concentration in this serum sample. Other inflammatory markers such as CRP, ferritin, procalcitonin, LDH, and D-dimer were measured in the serum sample collected on the first day of hospitalization. The concentration of the inflammatory biomarkers was correlated with the severity of COVID-19, the vaccination status, and the SARS-CoV-2 variant.

We quantified the IL-6 concentration by performing a chemiluminescence immunoassay, using the MAGLUMI IL-6 kits (Snibe Diagnostic, Shenzhen, China, Catalog number: 130616004M). We used the MAGLUMI 800 device (Snibe Diagnostic, Shenzhen, China) to assess the IL-6 concentrations. The assay is linear between 1.5 pg/mL and 5000 pg/mL.

### 4.3. Statistical Analysis

The statistical analysis was conducted using version 26 of the IBM SPSS software (Armonk, New York, NY, USA). We used the Kolmogorov–Smirnov test to assess the distribution of the variables, the Spearman correlation test to correlate the parameters, and group statistic tests to compare the inflammatory marker values between the vaccination status and the severity of COVID-19. A patient’s blood test results were compared with the reference interval of each parameter using a one-sample *t*-test. *p* < 0.05 was considered significant due to the α-significance level. The correlation ranking was established using the r results as follows: 0–0.29, poor correlation; 0.3–0.49, moderate correlation; 0.5–1, strong correlation. We used the ROC curve and area under the curve (AUC) to test the sensitivity and specificity of the inflammatory markers and vaccination status in predicting the risk of a severe form of COVID-19. Mean, median, standard deviation, and variance were calculated using SPSS and are listed for all investigated inflammatory markers. The results, discussions, and conclusions in the study are based on statistical analysis results.

### 4.4. Ethical Principles

The study complied with the ethical principles stated by the World Medical Association Declaration of Helsinki regarding medical research involving human subjects. The study was approved by the Commission of Ethics of Research from the University of Medicine and Pharmacy “Grigore T. Popa” Iași, Romania (IRB number: 99/2021), and by the Hospital Ethics Committee (IRB number: 30/2022).

## 5. Conclusions

Our findings confirmed the interdependent relationships observed between inflammation markers in relationship with the clinical severity of COVID-19, with all the tested biomarkers increasing in severe and critical COVID-19. IL-6, CRP, ferritin, LDH, and D-dimer were increased in all forms of COVID-19. These inflammation markers were lower in Omicron-infected patients. The unvaccinated patients developed more severe forms compared to the vaccinated ones, and a higher proportion of them needed hospitalization. D-dimer could predict a severe form of COVID-19, while LDH could predict the virus variant.

## Figures and Tables

**Table 1:** Group characteristics depending on the severity of COVID-19.

	Unit	Mild Form Number (%)	Moderate Form Number (%)	Severe Form Number (%)	Critical Form Number (%)	Reference Interval
Total Patients per Group	patients	30	49	28	30	
Men		16 (53.3%)	22 (44.9%)	20 (71.4%)	19 (63.3%)	
Vaccinated		15 (50%)	16 (32.7%)	4 (14.3%)	10 (33.3%)	
Delta virus Variant		8	22	18	12	
Omicron virus Variant		7	7	7	4	
The mean value of IL-6	pg/mL	11.96	25.46	78.03	458.31	0–7 pg/mL
The mean value of LDH	UI/L	198.28	292.52	365.63	464.60	100–210
The mean value of CRP	mg/L	32.74	52.54	112.32	124.93	0–5
The mean value of Ferritin	ng/mL	366.58	832.32	873.28	1591.273	25–350
The mean value of D-dimer	FEU/mL	0.59	0.74	1.18	1.67	0–0.5

**Table 2:** Spearman Correlation test on the tested biomarkers correlated by the severity of COVID-19, gender, and virus variant.

		IL-6	LDH	CRP	Ferritin	D-Dimer	Procalcitonin	The Severity of COVID-19
IL-6	*r*	1.000	0.484	0.519	0.430	0.456	0.315	0.624
	*p*		0.000	0.000	0.000	0.000	0.040	0.000
LDH	*r*	0.484	1.000	0.572	0.549	0.366	0.202	0.645
	*p*	0.000		0.000	0.000	0.000	0.211	0.000
CRP	*r*	0.519	0.572	1.000	0.489	0.489	0.451	0.537
	*p*	0.000	0.000		0.000	0.000	0.002	0.000
Ferritin	*r*	0.430	0.549	0.489	1.000	0.478	0.134	0.510
	*p*	0.000	0.000	0.000		0.000	0.422	0.000
D-dimer	*r*	0.456	0.366	0.489	0.478	1.000	0.117	0.416
	*p*	0.000	0.000	0.000	0.000		0.504	0.000
The severity of the disease	*r*	0.624	0.645	0.537	0.510	0.416	0.458	1.000
	*p*	0.000	0.000	0.000	0.000	0.000	0.002	

IL-6—interleukin-6; CRP—C-reactive protein; LDH—lactate dehydrogenase; highlighted *p* values are statistically significant.

**Table 3:** Significant differences in tested biomarkers depending on the severity form.

Biomarkers	Mild vs. Moderate	Moderate vs. Severe	Severe vs. Critical
IL-6	*p* > 0.05	*p* < 0.05	*p* < 0.05
CRP	*p* > 0.05	*p* < 0.05	*p* > 0.05
Ferritin	*p* < 0.05	*p* > 0.05	*p* > 0.05
LDH	*p* > 0.05	*p* > 0.05	*p* < 0.05
D-dimer	*p* > 0.05	*p* > 0.05	*p* > 0.05
Procalcitonin	*p* > 0.05	*p* > 0.05	*p* > 0.05

Highlighted *p* values are statistically significant.

**Table 4:** Mean, median, standard deviation, and variance of the tested biomarkers.

	IL-6	LDH	CRP	Ferritin	D-Dimer	Procalcitonin
Mean	125.64	329.18	76.8	1073.87	1.02	1.72
Median	19.71	279	49.07	485.75	0.55	2.00
Std. Deviation	485.83	204.22	76.97	2294.28	1.48	0.8
Variance	236,036.27	41,707.83	5925.01	5,263,722.81	2.17	0.64

IL-6—interleukin-6; CRP—C-reactive protein; LDH—lactate dehydrogenase.

**Table 5:** Comparison of the IL-6 mean value with the results obtained by other authors, depending on COVID-19 severity (pg/mL).

Authorship	Mild Form	Moderate Form	Severe Form	Critical Form	Reference
Our current results	11.96	25.46	78.03	458.31	
Sun et al.	5.26 ± 1.25	14.17 ± 11.37	33.22 ±31.9	34.09 ± 26.47	[13]
Liu et al.		39.3 ± 71.1	55.6 ± 44.4	81.4 ± 65.6	[14]
Zheng et al.		27.6	64.3		[15]
He et al.		14	14.3		[16]
Liu et al.	7.1		29.1		[17]

**Table 6:** Comparison of the D-dimer mean value with the results obtained by other authors, depending on COVID-19 severity (µg/mL).

Authorship	Mild Form	Moderate Form	Severe Form	Critical Form	Reference
Our current results	0.3	0.37	0.6	0.84	
Sun et al.	0.16 ± 0.06	0.35 ± 0.25	3.15 ± 3.31	1.95 ± 2.38	[13]
Liu et al.		0.74 ± 2.28	1.17 ± 4.27	4.138 ± 7.5	[14]
Zheng et al.		0.24	1		[15]
He et al.		0.32	0.95		[16]
Liu et al.	0.26		1		[17]
Zou et al.	0.43	-	1.04		[21]
Liu et al.	0.4	-	0.9		[22]
Shang et al.		0.51	0.84		[23]
Chen et al.		0.3	2.6		[24]
Güner et al.	0.33		0.95		[25]

**Table 7:** Comparison of the ferritin mean value with the results obtained by other authors, depending on COVID-19 severity (ng/mL).

Authorship	Mild Form	Moderate Form	Severe Form	Critical Form	Reference
Our current results	366.58	832.32	973.28	1591.27	
Liu et al.	367.8		835.5		[22]
Chen et al.		337.4	1598		[24]
Güner et al.	96		433		[25]
Itelman et al.	263.6	769.2	579.8		[26]
Wang et al.	821.1		1331	1368	[27]
Liu et al.		155.70	827.2		[28]

**Table 8:** Comparison of the LDH mean value with the results described by other authors, depending on COVID-19 severity (UI/L).

Authorship	Mild Form	Moderate Form	Severe Form	Critical Form	Reference
Our current results	198.28	292.52	365.63	464.63	
Sun et al.	231.75 ± 122.92	217.47 ± 51.12	279.70 ± 84.26	376.89 ± 161.55	[13]
Zheng et al.		194	247		[15]
He et al.		188	276		[16]
Liu Y et al.	221		383		[17]
Liu J et al.	221.5		462.4		[22]
Shang et al.		206	277		[23]
Chen et al.		224	537		[24]
Güner et al.	209		308		[25]
Itelman et al.	298	367.5	538		[26]
Wang et al.	305.6		424.1	542.5	[27]

**Table 9:** Comparison of the CRP mean value with the results described by other authors, depending on COVID-19 severity (mg/L).

Authorship	Mild Form	Moderate Form	Severe Form	Critical Form	Reference
Our current results	32.74	52.55	112.32	123.93	
Sun et al.	5.08 ± 8.58	11.83 ± 17.07	48.92 ± 72.71	30.96 ± 29.46	[13]
Liu et al.		37 ± 43.4	61.5 ± 63.8	75 ± 59.4	[14]
Zheng et al.		6.14	29.9		[15]
Liu et al.	7.6		62.9		[22]
Shang et al.		10.05	43.15		[23]
Chen et al.		22	139.4		[24]
Itelman et al.	68.1	98.5	132.4		[26]
Wang et al.	53.6		91.8	114.9	[27]

## Data Availability

The data presented in this study are available on request from the corresponding author. The data are not publicly available due to ethical reasons.

## Supplementary Materials

The following supporting information can be downloaded at: https://www.mdpi.com/article/10.3390/ijms24108550/s1.
