# Supplementary material for: Inflammatory Response in COVID-19 Depending on the Severity of the Disease and the Vaccination Status
Source: Int J Mol Sci. 2023 May 10;24(10):8550. doi: 10.3390/ijms24108550 (PMC10218522; doi:10.3390/ijms24108550)
Supplement: Supplementary file 1 [file ijms-24-08550-s001.zip › ijms-2388527-supplementary.pdf]

Supplementary Materials

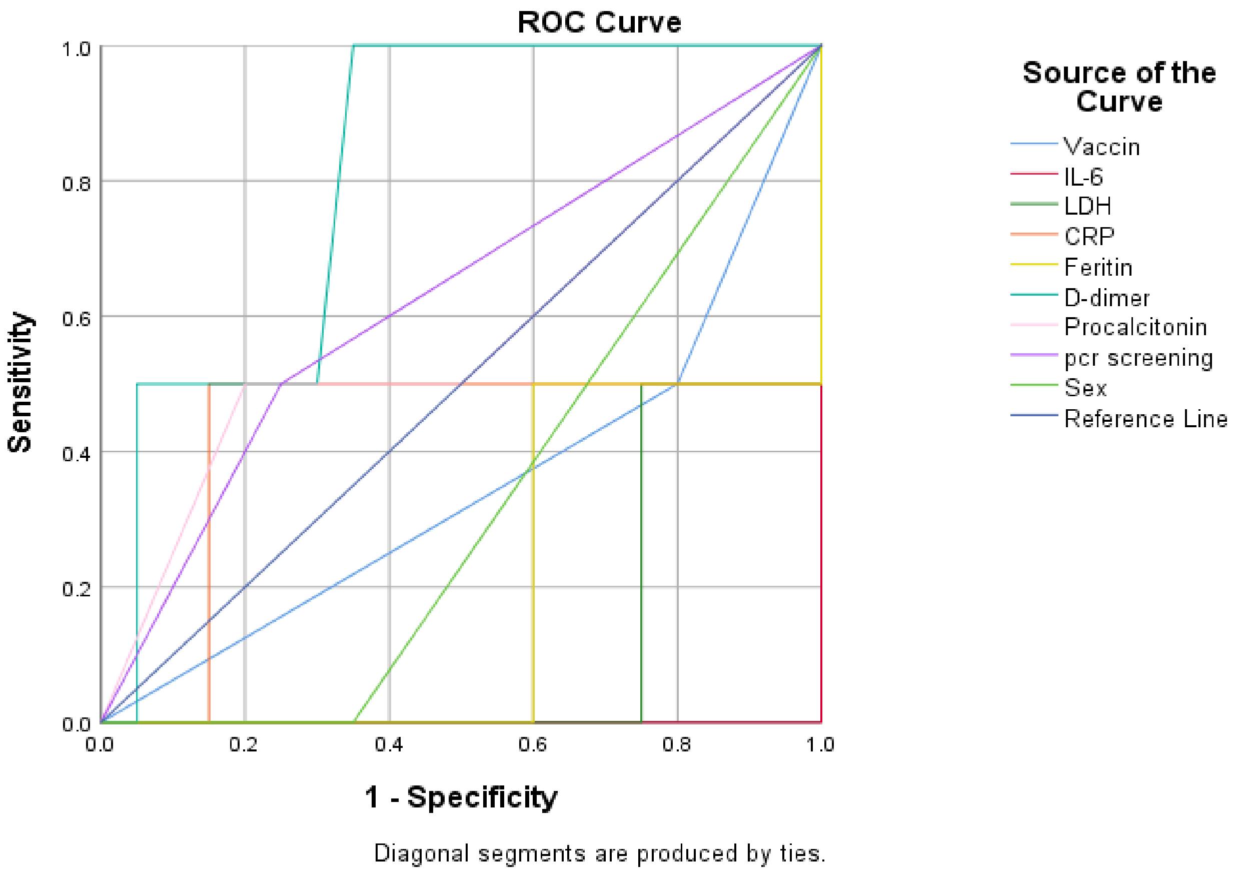

Supplementary figure S1. ***ROC curves generated for biomarkers that best predicted the form of COVID-19:D-dimer.*** *D-dimer predicted the infection severity in our study group. For this analysis, the larger values of the test variables indicate stronger evidence of a positive actual state (severity of COVID-19), while the AUC values between 0.7-0.8 define a good capacity of discrimination.*

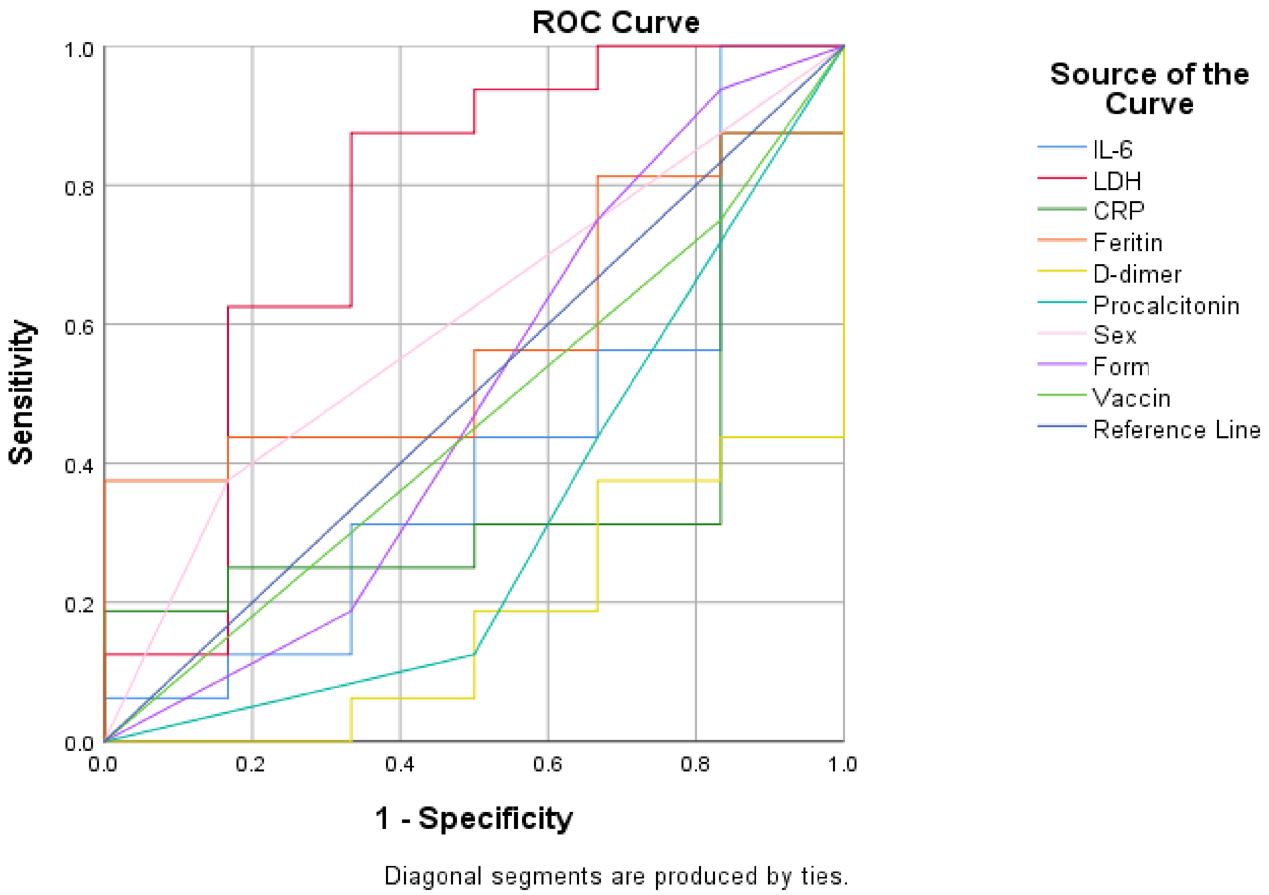

Supplementary figure S2. ***ROC curves generated for biomarkers that best predicted the SARS-CoV-2 variant: LDH.*** *LDH predicted the virus variant in our study group. For this analysis, the larger values of the test variables indicate stronger evidence of a positive actual state (Omicron variant), while the AUC values between 0.7-0.8 define a good capacity of discrimination.*
